# Integrative functional profiling of rhizospheric and endophytic bacteria reveals *Lelliottia aquatilis* as a multifunctional PGPR in Himalayan ecosystems

**DOI:** 10.3389/fsysb.2026.1872892

**Published:** 2026-08-03

**Authors:** Shikha Devi, Riya Chandel, Damini Thakur, Shweta Pathania, Simran Chauhan, Simran Simran, Nishtha Pathak, Poonam Katoch, Taniya Tyagi, Sushila Devi, Arvind Kumar Bhatt, Sveta Thakur

**Affiliations:** 1 Department of Microbiology, Himachal Pradesh University, Shimla, Himachal Pradesh, India; 2 Department of Biosciences, Himachal Pradesh University, Shimla, Himachal Pradesh, India; 3 Department of Biotechnology, Himachal Pradesh University, Shimla, Himachal Pradesh, India

**Keywords:** abiotic stresses, biocontrol, biofertilizers, Endophytes, PGPR, phosphate solubilization, sustainable agriculture, termite mound associated plants

## Abstract

**Background:**

Plant growth-promoting (PGP) bacteria have traditionally been known to have the ability to enhance crop productivity, abiotic stress-tolerance and disease resistance and providing an environmentally-friendly alternative to the currently used chemical fertilizers and pesticides. In this study, endophytic and rhizospheric bacteria were isolated from *Myrica esculenta* and plants residing in termite mounds, i.e., *Carissa carandas* and *Pyrus pashia*, and analyzed for PGP properties, resistance to abiotic stress and biocontrol capabilities.

**Methods:**

Bacterial isolates were screened thoroughly for PGP activities, biocontrol inhibition, biofilm production, extracellular polymeric substance production and catalase activity. Selected isolates were further tested for stress tolerance (salinity, drought and pH extremes), seed germination and growth promotion. The most efficient isolate was subjected to molecular characterization using 16S rRNA sequencing.

**Results:**

Seven isolates reliably displayed a wide range of PGP characteristics: efficient phosphate solubilization (up to 12.8 µg/mL), high indole-3-acetic acid (IAA) levels (up to 16.8 µg/mL), siderophore activity and strong ammonia release. Fungal pathogens, i.e., *Fusarium oxysporum, Fusarium solani* and *Macrophomina phaseolina* were substantially inhibited by *Lelliottia aquatilis* strain MC3 in biocontrol experiments. Stress -tolerance tests showed strong tolerance to salt (up to 5% NaCl, survival at 10% NaCl), drought (PEG 25%) and pH extremes (5–9). Chickpea growth assays confirmed its functional relevance, with inoculation of MC3 achieving the highest shoot length enhancement. Molecular identification revealed MC3 as *L*. *aquatilis*, a taxon rarely reported as plant growth promoting rhizobacteria (PGPR).

**Conclusion:**

These potential isolates are considerable candidates for biofertilizers and biocontrol developments in stress prone environments. *L*. *aquatilis* strain MC3 emerged as the most promising isolate due to its multifaceted PGP traits, strong biocontrol efficacy, abiotic stress-tolerance, and proven plant growth enhancement. To our knowledge, this is one of the first reports for identification of *L*. *aquatilis* as multifunctional PGPR from Himalayan ecosystems. This study introduces *L*. *aquatilis* strain MC3 as an emerging candidate for bioinoculant development.

## Graphical Abstract

**Figure:**
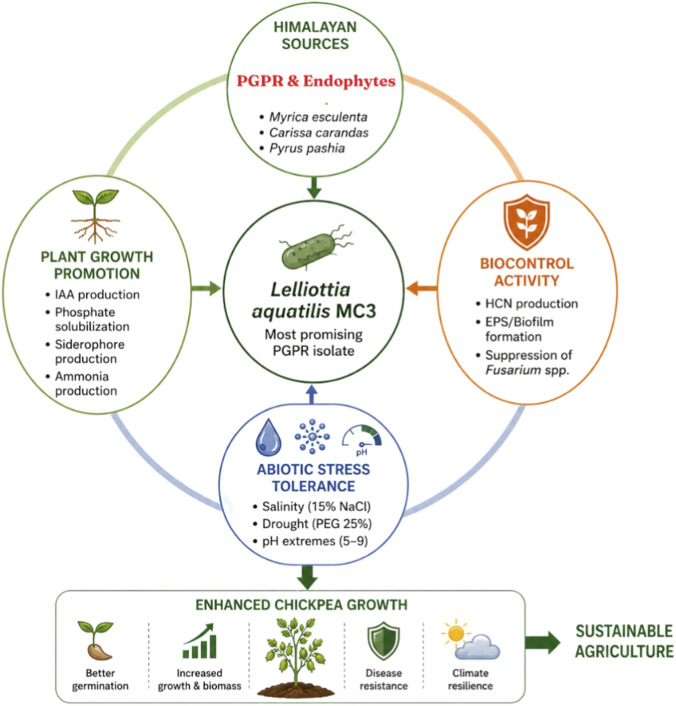

## 1 Introduction

The growing need to adopt environmentally benign agricultural practices has provoked the intensive research of PGPR and endophytic microorganisms as the alternative to synthetic fertilizers and pesticides. Endophytes and PGPR are now acknowledged as vital elements of a resilient agricultural system, not just as supplements. Besides simply increasing yield, their multifunctional qualities also include lowering abiotic stressors like salinity, drought, and pH imbalances, which will only become more noticeable offered the possible effects of climate change (Mellidou and Karamanoli, 2022). Therefore, these microbial resources are essential for climate-adaptive agroecosystems that can sustain productivity in the face of growing environmental constraints.

The rhizosphere is a small but dynamic area of soil that surrounds plant roots. It acts as a vital hot spot for intricate biochemical and ecological interactions between plants, microbes, and soil components. In this niche, the PGPR have taken a central role in determining the plant health and productivity. PGPR enhance plant growth directly through solubilization of nutrients and phytohormones production and indirectly through pathogen suppression and induced systemic resistance (Wahab et al., 2024). Similarly, endophytic bacteria promote plant growth by enhancing nutrient uptake, stress tolerance and pathogen resistance without causing disease (Santoyo et al., 2016; Tariq et al., 2025). As a result, these microbial communities offer a useful and diverse arsenal for sustainable agriculture.

Among the diverse attributes demonstrated by PGPR, phosphate solubilization has attracted the highest attention in research. Phosphorus, which is one of the most important macronutrients, is often immobilised in soil in insoluble forms hence reducing its availability to plants. The presence of bacteria, which can release organic acids and phosphatases, transforms insoluble phosphates into soluble forms, which are assimilated by the root and lead to increased crop production (Andrade et al., 2023). Recent findings highlight that phosphate-solubilizing PGPR are crucial for lowering reliance on chemical fertilizers and improving nutrient utilization efficiency in sustainable agricultural systems (Wahab et al., 2024). Another key mechanism is the synthesis of IAA, a phytohormone which improves the elongation and branching of roots, increasing the absorptive surface area. PGPR that produce IAA also help plants withstand abiotic stress by enhancing root plasticity and resilience (Ayele et al., 2021; Andrade et al., 2023).

Siderophore production is an example of the versatility of the PGPR. The siderophores do not only aid plants in obtaining this necessary micronutrient by chelating iron in a limiting environment, but they also inhibit pathogens by withholding the necessary resources (Compant et al., 2019; Falcon-Pineiro et al., 2026). Production of hydrogen cyanide and ammonia also expands the range of microbes, leading to the inhibition of pathogens and enrichment of nutrients. Catalase activity prevents bacteria from oxidative stress. Biofilm formation and extracellular polymeric materials enhance root colonization and bacterial tolerance to unfavorable environmental conditions. These processes work together to enable PGPR to support plant defense and growth (Sungthongwises et al., 2025; Giri and Virk, 2025).

Worldwide, damage to crops is caused by fungal soil pathogens such as *F. oxysporum*, *F. solani*, and *M. phaseolina*. The use of chemical fungicides, which are harmful to the environment and human health, is the foundation of traditional management techniques. The production of antifungal metabolites, nutrient competition, and fungal hyphae morphological deformity by PGPR and endophytes are the sustainable alternatives. Microscopic observation of fragmented, degenerated hyphae with broken cell walls in the presence of PGPR highlights their antagonistic potential (Aslam et al., 2025; Chhetri et al., 2025).

Himalayan region, which is diverse in flora and agro-climatic conditions, is an unexplored pool of microbial diversity. It experiences a mean annual rainfall of 1,000–1800 mm, strong seasonal variations in temperature (−5 °C in winter to approximately 30 °C in summer), and an increasing prevalence of drought episodes due to climate change. Additionally, soils are often nutrient poor and erosion prone due to steep slopes, resulting in fragile ecosystems (Matta et al., 2025). *M*. *esculenta*, a medicinally as well as commercially important tree species, grows in soils of low nutrient content, implying that it has a strong relationship with efficient rhizospheric microorganisms. However, termite mounds are microhabitats that are highly rich in organic contents and microbial communities, which are adapted to stressful conditions. Endophytes and rhizospheric bacteria with unique functional traits are also likely to be present in plants that grow near termite mounds, such as *C*. *carandas* and *P*. *pashia* (Devi and Thakur, 2018). Examining these distinct ecosystems can reveal details about functional diversity and microbial adaptation. Microbes associated with medicinal plants and the termite mound ecosystems are less studied compared to the use of agricultural soils to produce PGPR. Additionally, these niches may contain strains that are more adaptable and resilient (Sungthongwises et al., 2025). Such strains could offer new platforms for the development of biofertilizer and biocontrol applications.

To exploit these resources, morphological, biochemical, and molecular characterization of promising PGPR is crucial for achieving taxonomic clarity and for emphasizing unique microbial resources (Lotfi et al., 2022)**.** While the well-known PGPR genera include *Bacillus*, *Pseudomonas*, *Rhizobium*, *Azotobacter*, *Azospirillum*, *Klebsiella*, *Arthrobacter*, *Burkholderia*, *Alcaligenes,* and *Enterobacter* (Devi et al., 2023; Lotfi et al., 2022), the finding of *L*. *aquatilis* as a potential PGPR expands the range of existing genera and opens up new possibilities in biofertilizer development.

Microbial survival and efficacy in agroecosystems of stress require abiotic stress-tolerance. Saline, drought, and pH-tolerant isolates have greater chances of being useful in the field. These stress-tolerant PGPR can survive under unfavorable conditions and also maintain their PGP and biocontrol activities. Their acceptance in climate-sensitive agriculture also necessitates this strength as environmental variability is always an unending challenge (Joshi et al., 2023).

Considering these factors, the current study was conducted to isolate and characterize rhizospheric bacteria associated with *M*. *esculenta* as well as endophytic and rhizospheric bacteria from termite-mound associated plants. The isolates were evaluated for their PGP properties, biocontrol activities against important fungal pathogens, impacts on plant growth, and tolerance to abiotic stresses. Promising isolates were further identified by morphological, biochemical and molecular characterization studies. This research will combine findings of two different ecological niches and thus help to find multifunctional bacterial isolates which can be used as biofertilizers and biocontrol agents. This will largely help in achieving sustainable agriculture in stressful environments.

## 2 Materials and methods

### 2.1 Sample collection

Rhizospheric soil samples were collected from the roots of *M*. *esculenta* plants from three locations in Shimla, Himachal Pradesh, India (Supplementary Figure S1). Rhizospheric soil from 10–15 cm depth was aseptically collected in sterile containers. Soil and plant samples from termite mounds were collected from *C*. *carandas* and *P*. *pashia* plants located in the Solan District of Himachal Pradesh, India (Supplementary Figure S1). Samples were collected during January and February 2025. At each site, three random samples of rhizospheric soil from different locations were pooled to obtain a composite sample. In the case of leaf and stem tissues, three samples were aseptically excised using sterile scissors and thoroughly mixed to form a composite sample for endophytic bacterial isolation. Supplementary Table S1 displays the geographical locations of sampling sites and details of the collected samples. The samples were transported aseptically and analysed within 24 h to ensure microbial survival (Gavande et al., 2024).

*M*. *esculenta* rhizospheric samples were collected from Shimla district, and rhizospheric and endophytic samples from termite-mound-associated plants were collected from Solan district of Himachal Pradesh, India. Both districts are part of the Western Himalayan ecosystem, which is distinguished by monsoon-dominated rainfall and significant seasonal temperature variation (from roughly 2 °C in winter to 30 °C in summer). The annual precipitation in Shimla is about 1,530 mm and in Solan it ranges from 1,200 to 1,400 mm, with most rainfull occurring during the monsoon season. Soils are generally slightly acidic to neutral, low in salinity and often nutrient deficient (Ali et al., 2026; Kumar et al., 2025; Pandey et al., 2022). Furthermore, the unique physicochemical properties of termite mounds and the temporary moisture limitation form unique ecological niches that might be beneficial to the selection of stress-tolerant microorganisms (Devi and Thakur, 2018; Sungthongwises et al., 2025).

### 2.2 Isolation of rhizospheric and endophytic bacteria

Rhizospheric bacteria were isolated by the serial dilution plate method on nutrient agar (NA) (Gavande et al., 2024). Morphologically different colonies were streaked for purification. Endophytic bacteria were isolated from surface sterilized stems and leaves of termite mound plants. Plant segments were disinfected with 70% ethanol followed by 2% sodium hypochlorite solution and subsequenty washed several times with sterile distilled water. To confirm the efficiency of surface sterilization, 100 μL of the final rinse water was plated on NA plates and incubated at 30 °C for 24–48 h. The absence of microbial growth confirmed the successful surface sterilization (Gavande et al., 2024). Subsequently, the sterile tissues were aseptically macerated and plated on NA for isolation of bacteria. NA was selected as a general-purpose medium because it supports the growth of a wide range of culturable heterotrophic bacteria. The medium consisted of peptone (5 g/L), yeast extract (3 g/L), sodium chloride (5 g/L), and agar (15 g/L), with a final pH of 7.0 ± 0.2. Plates were incubated at 30 °C ± 2 °C for 24–48 h. Pure cultures were obtained by subculturing distinct colonies through repeated streaking and were subsequently stored on NA slants at 4 °C for further study.

### 2.3 Screening for PGP traits

#### 2.3.1 Phosphate solubilization

Bacteria were grown on Pikovskaya’s (PVK) agar at 30 °C ± 0.1 °C for 5 days. Additionally, uninoculated PVK agar plates served as the negative control. Solubilization was indicated by halo formation (Mayadunna et al., 2023) and the phosphate solubilization index (PSI) was determined as:

Phosphate solubilization index (PSI) = (Colony diameter + Halo zone diameter)/Colony diameter.

Quantitative assessment was done in PVK broth with 0.5% tricalcium phosphate (TCP) using vanadomolybdophosphoric acid reagent, measured at 430 nm (Suleiman et al., 2026).

#### 2.3.2 IAA production

Qualitative estimation was performed in nutrient broth containing 1% tryptophan, incubated at 30 °C ± 0.1 °C for 48 h in the dark. Uninoculated broth containing 1% L-tryptophan served as the negative control. The supernatant was mixed with Salkowski’s reagent, and a pink color was observed in the presence of IAA. Quantitative estimation was done with Salkowski’s reagent and absorbance at 535 nm. Pure IAA was used to prepare a standard curve (Chhetri et al., 2025).

#### 2.3.3 Hydrogen cyanide (HCN) production

HCN Production was assessed on NA with added glycine and picrate-impregnated filter paper. Uninoculated glycine-amended medium served as the negative control. The plates were incubated at 28 °C ± 0.1 °C for 4–5 days and a change in color indicated HCN production (Devi et al., 2023; Sehrawat et al., 2022).

#### 2.3.4 Siderophores production

Siderophore activity was tested using the chrome azurol S (CAS) assay, a universal colorimetric method, which is independent of the structure of the siderophores. Siderophores bind iron from the Fe CAS hexadecyltrimethylammonium bromide complex, releasing CAS dye and resulting in a color change from blue to orange (Chhetri et al., 2025; Singh et al., 2020). Additionally, uninoculated CAS agar plates served as the negative control.

#### 2.3.5 Ammonia production

Ammonia production was measured in peptone water using Nessler’s reagent (Devi et al., 2023; Singh et al., 2020). Uninoculated peptone water served as the negative control. A yellow to brown colour was observed after the addition of 0.5 mL of the reagent.

#### 2.3.6 Bacterial biofilms and exopolysaccharides (EPS) production

The tube adherence method was used to assess biofilm production (Gogoi et al., 2025). Isolates were cultured in tryptic soy broth (TSB) supplemented with 1% glucose at 30 °C for 24–48 h. Uninoculated broth served as the negative control. The tubes were washed, stained with crystal violet (0.1%), rinsed, and dried. Positive biofilm formation was indicated by biofilm rings on tube walls. EPS production was determined by growing isolates in nutrient broth at 30 °C ± 2 °C for 5 days (Gogoi et al., 2025). Cultures were centrifuged and filtrates were treated with cold ethanol (1:1). Formation of white precipitates indicated the EPS production.

#### 2.3.7 Catalase activity

Catalase activity was determined by adding three to four drops of H_2_O_2_ directly to bacterial colonies. Positive catalase activity was indicated by immediate effervescence (Devi et al., 2023).

#### 2.3.8 Biocontrol assays

Antagonism was evaluated against *F. oxysporum*, *F. solani* and *M*. *phaseolina* using dual culture technique on PDA and NA (1:1) (Ayele et al., 2021; Gogoi et al., 2025). A 5 mm mycelial plug from an actively growing fungal culture was placed at the center of the plate, and the bacterial isolate was streaked approximately 3 cm from the fungal plug. Control plates contained only the mycelial plug. Inoculated plates were incubated at 28 °C ± 2 °C for 7 days and the percentage growth inhibitions (PGIs) were recorded (Pallavi et al., 2023). PGI was calculated using the following formula:

$$\text{PGI }\left(\%\right)=\left[\left(C\;-\;T\right)\;/\;C\right]*100$$

Where, C is growth of the pathogen in the control plate and T is growth of the pathogen in the dual culture plate. All treatments were performed in triplicate and results were expressed as mean percentage inhibition values. For microscopic analysis, fungal hyphae were stained with lactophenol picric acid mounting reagent and examined under a microscope to check for morphological changes, including swelling, fragmentation and degeneration (Safni and Antastia, 2018). Additionally, representative images of the dual culture assays were assembled solely for presentation purposes. No image manipulation affecting the scientific interpretation of the results was performed. Original unprocessed images are provided in Supplementary Data S2.

### 2.4 Abiotic stress-tolerance assays

Bacterial tolerance to salinity, pH and drought stress was assessed by measuring growth at optical density (OD) 600 nm. While OD measurements allow a rapid assessment of bacterial growth under stress conditions, CFU-based analysis would provide a better estimate of bacterial survival and may be considered in future studies.

#### 2.4.1 Drought resistance

*In vitro* water stress-tolerance of the bacterial isolates was tested. Nutrient broth with varying water potential (10%, 15%, 20% and 25%) was prepared by supplementing the broth with the required amount of polyethylene glycol (PEG 6000) (Merck). Different concentrations of PEG 6000 were used to simulate progressive drought stress and select bacterial isolates capable of tolerating water limiting conditions that occur in agricultural environments. The culture was inoculated with 1% overnight bacterial culture in the nutrient broth and incubated at 30 °C ± 0.1 °C for 24 h, and OD was measured at 600 nm (Devi et al., 2023).

#### 2.4.2 Salt tolerance

Salt tolerance of bacterial isolates was determined by inoculating bacterial culture into nutrient broth containing different concentrations of NaCl (0.5%–10%) (Devi et al., 2023).

#### 2.4.3 pH tolerance

To determine the survival of rhizobia under different pH conditions, each strain was inoculated into nutrient broth with different pH (5, 6, 7, 8 and 9). pH 6 and below was considered acidic and pH 8 and above alkaline. The replicate cultures were incubated at 30 °C for 24 h and OD was measured at 600 nm (Amna et al., 2020).

### 2.5 Plant growth enhancement assay

Chickpea (*Cicer arietinum*) is a globally important pulse selected as the model crop due to its agronomic relevance in the Himalayan region and its strong responsiveness to PGPR inoculation. Based on multifarious PGP characteristics and abiotic stress-tolerance, isolates MC3, SR2 and K1III were selected for plant growth enhancement assay. To evaluate the efficacy of bioformulation treatments, four experimental groups were established: (i) the control group, (ii) Treatment 1 (T1) with inoculation of MC3, (iii) Treatment 2 (T2) with inoculation of SR2 and (iv) Treatment 3 (T3) with inoculation of K1III.

In the present study, chickpea seeds were purchased from local markets in Shimla, Himachal Pradesh, India. Chickpea seeds were surface sterilized with 70% ethanol and washed with sterile distilled water. Bacterization of sterilized seeds was done as the method described by Devi et al. (2023). Selected bacterial isolates were cultured in nutrient broth at 120 rpm, 28 °C for 48 h prior to seed bacterization. The culture was centrifuged at 7,100 *g* for 15 min at 4 °C to get the pellet, washed with sterile distilled water, and resuspended to a concentration of 10^8^ CFU/mL. The slurry was prepared by mixing the suspension with 1% carboxymethyl cellulose (CMC) and applied to the surface of sterilized seeds. The seeds were dried in an aseptic environment, transferred to plastic pots, and sown at a depth of 2 cm. Growth observations were recorded at 7 and 14 days after sowing, and all experiments were carried out in duplicate pots filled with non-sterile soil under natural photoperiod conditions. The pots were watered every other day to maintain adequate soil moisture and to mimic natural growth conditions. Furthermore, statistical methods were used to determine significance between selected treatments, including one-way ANOVA followed by Tukey’s *post hoc* test at *p* < 0.05.

### 2.6 Identification of potential bacterial isolates

#### 2.6.1 Morphological and biochemical characterization

Potential bacterial isolates were identified using various biochemical tests and Bergey’s Manual of Systematic Bacteriology was used as a source of identification keys to identify promising bacterial isolates (Islam et al., 2016).

#### 2.6.2 Molecular characterization of potential bacterial isolate

The most promising isolate MC3 was chosen for molecular analysis. Genomic DNA was isolated and the 16S rRNA gene was amplified with universal primers (Das et al., 2024). The sequences were searched against the NCBI GenBank database using BLAST (Devi et al., 2023; Das et al., 2024). Phylogenetic analyses were performed using MUSCLE alignment and MEGA11 (Divyanshu et al., 2022). The accession number was obtained by submitting the sequence to GenBank and the accession number assigned to bacterial isolate MC3 (*Lelliottia aquatilis*) is PZ232191.

### 2.7 Statistical analysis

All experiments were performed in triplicate and the values presented were the mean values of triplicate. The data obtained after the experiments were statistically evaluated using one way ANOVA with a Tukey *post hoc* test using Prism5 software (GraphPad, La Jolla, CA, USA). Means with different lowercase letters differ significantly when the calculated p-values were p < 0.05.

## 3 Results

### 3.1 Isolation of endophytic and rhizospheric bacterial isolates

A total of twenty-seven bacterial isolates were successfully obtained from the rhizosphere of *M*. *esculenta* and termite mound–associated plants (*C*. *carandas* and *P*. *pashia*). Of these, nine isolates (MC1–MC9) were recovered from the rhizosphere of *M*. *esculenta*, ten isolates (SR1–SR10) from the rhizospheric soils of termite mound–associated plants, and eight endophytic isolates (K1I–K1IV, K2I–K2IV) from plant tissues. The description of bacterial isolates obtained from different collection sites is provided in Table 1.

**TABLE 1:** Description of bacterial isolates obtained from different collection sites.

S. No.	Plant name	Location	Sample type	No. of bacterial isolates	Isolate codes
1	*M*. *esculenta*	Shimla district	Rhizospheric soil 1	3	MC1, MC2, MC3
			Rhizospheric soil 2	2	MC4, MC5
			Rhizospheric soil 3	4	MC6, MC7, MC8, MC9
2	*C*. *carandas*	Solan district	Rhizospheric soil	6	SR1, SR2, SR3, SR4, SR5, SR6
			Leaves	2	K1I, K1II
			Stem	2	K1III, K1IV
3	*P*. *pashia*		Rhizospheric soil	4	SR7, SR8, SR9, SR10
			Leaves	2	K2I, K2II
			Stem	2	K2III, K2IV

### 3.2 PGP activity of rhizobacteria as well as endophytic bacteria

**FIGURE 1:**
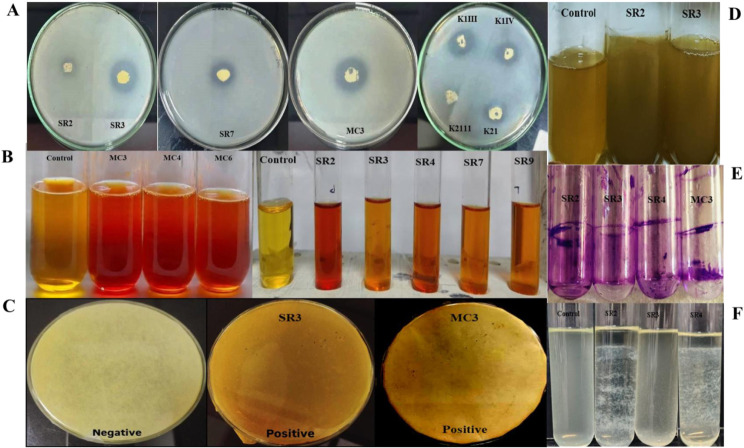
Screening of rhizospheric and endophytic bacterial isolates for various PGP attributes **(A)** Phosphate solubilization **(B)** IAA production **(C)** HCN production **(D)** Ammonia production **(E)** Biofilm production **(F)** EPS production.

The rhizospheric and endophytic bacterial isolates were tested for PGP activities through different qualitative and quantitative assays. The results of the different PGP attributes assessed in the present study are presented in Figure 1, including phosphate solubilization (Figure 1A), IAA production (Figure 1B), HCN production (Figure 1C), ammonia production (Figure 1D), biofilm formation (Figure 1E), and EPS production (Figure 1F).

#### 3.2.1 Phosphate solubilization

##### 3.2.1.1 Qualitative estimation of phosphate solubilization activity

A total of twenty seven bacterial isolates (ninteen rhizospheric and eight endophytic) were screened for phosphate solubilization on PVK agar (Figure 1A; Table 2). Clear halo zones surrounding colonies confirmed their ability to solubilize insoluble phosphate. Out of the total isolates, only 55.56% bacterial isolates were found to be phosphate solubilizing. Among rhizospheric isolates, MC3, SR3, SR7 and SR2 were the most efficient, with solubilization indices of 3.30, 3.10, 2.50 and 2.25, respectively. Endophytic isolates K1III and K1IV also showed notable activity, with indices of 2.60 and 1.90, respectively. Overall, rhizospheric isolates exhibited a higher propensity for phosphate solubilization than endophytic ones.

**TABLE 2:** Combined PGPR traits of rhizospheric and endophytic bacterial isolates.

Isolate	Phosphate solubilization index	IAA (µg/mL)	HCN	Siderophore	Ammonia	Biofilm	EPS	Catalase
MC1	1.05^a^	+	–	+	–	–	–	Positive
MC3	3.30 ^d^	+++	++	++	+	+	+	Positive
MC4	1.35^a^	+++	+	+	+	–	+	Positive
MC6	1.17^a^	+++	+	++	+	–	–	Positive
MC8	1.08^a^	++	–	–	–	–	–	Positive
MC9	1.12^a^	++	–	–	–	–	–	Positive
SR2	2.25 ^b^	+++	–	+	++	++	++	Positive
SR3	3.10 ^d^	+	++	+	++	+	+	Positive
SR4	1.25^a^	+	–	–	–	++	++	Positive
SR7	2.50^c^	+++	–	+	–	–	–	Positive
SR9	1.20^a^	+	–	–	–	–	–	Positive
K1III	2.60^c^	+++	+	+	+	+	+	Positive
K1IV	1.90 ^b^	++	–	–	–	+	+	Positive
K2I	1.40^a^	+++	–	–	–	–	–	Positive
K2III	1.10^a^	++	–	+	+	+	+	Positive

–=no activity;+= weak activity; ++=moderate activity, +++=strong activity. Means with different lowercase letters in the same column differ significantly atp<0.05 according to Tukey *post hoc* test.

##### 3.2.1.2 Quantitative estimation of phosphate solubilization activity

**FIGURE 2:**
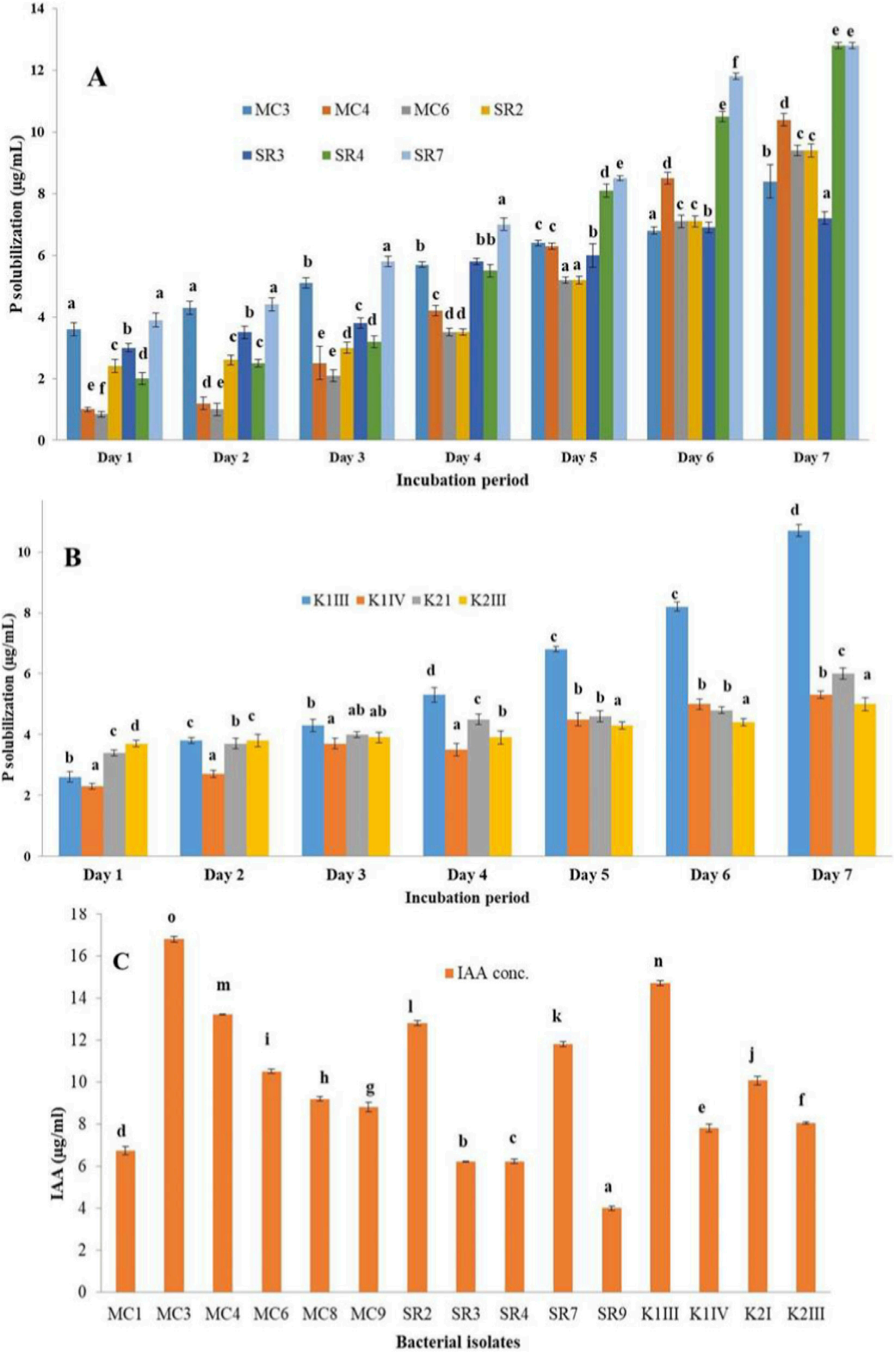
Phosphate solubilization by potential isolates in PVK broth **(A)** rhizospheric and **(B)** endophytic bacteria. **(C)** Comparison of IAA production across different bacterial isolates. Values are expressed as mean ± SD. Means with different lowercase letters above bars indicate statistically significant differences within the same day at p < 0.05, according to Tukey’s *post hoc* test.

Quantitative analysis of phosphate solubilization was conducted in PVK broth medium, with soluble phosphate levels measured from day 1 to day 7 of incubation (Figures 2A,B). Maximum solubilization was observed on the seventh day, with values ranging between 7.2 µg/mL and 12.8 µg/mL (Figures 2A,B). Among them, SR4 and SR7 recorded the highest solubilization values (12.8 µg/mL), followed by MC4 (10.4 µg/mL), and SR2 and MC6 (9.4 µg/mL). MC3 and SR2 showed moderate activity, while SR3 displayed comparatively lower solubilization.

Notably, the greatest halo zones in qualitative screening were found for MC3, SR3, SR7 and SR2, but the highest soluble phosphorus concentrations in broth culture were found for SR4 and SR7, implying discrepancy between agar-based and broth-based evaluations of phosphate-solubilizing activity.

Similarly, K1III was highly efficient, releasing 10.7 µg/mL by day 7. K2I, K1IV and K2III exhibited the moderate phosphate solubilization activity, reaching 6.0 µg/mL, 5.3 µg/mL and 5.0 µg/mL, respectively, with relatively stable phosphate concentrations. These findings highlight K1III as the most efficient endophytic phosphate solubilizer, while K2I and K2III may serve as moderate contributors to phosphate availability.

The pH of the broth declined from its initial value of 7.0 to 4.0–4.5 in efficient isolates (Supplementary Figures S2a, S2b). Among rhizospheric strains, SR4 and SR7 recorded the highest solubilization (12.8), with noticeable pH reductions (pH 5.5 and 4.5, respectively). MC6 and SR2 also showed strong solubilization (9.4), accompanied by noticeable reductions in pH to 4.4 and 4.0, respectively), while MC3 and MC4 demonstrated moderate solubilization. Endophytic isolates showed moderate pH reductions. K1III was the most effective (10.7, pH 5.5), whereas K2I and K2III displayed stable though lower solubilization.

#### 3.2.2 IAA production

##### 3.2.2.1 Qualitative estimation

Phosphate-solubilizing isolates were screened for IAA production, and all demonstrated positive activity, confirmed by the development of pink coloration in assay tubes (Figure 1B).

##### 3.2.2.2 Quantitative estimation

Quantitative analysis revealed varying levels of IAA production across potential rhizospheric and endophytic isolates (Table 2; Figure 1B; Supplementary Figure S3). Among rhizospheric isolates, MC3 (16.8 µg/mL), MC4 (13.2 µg/mL) and SR2 (12.8 µg/mL) produced comparatively higher levels. Endophytic isolates also showed significant activity, with K1III (14.7 µg/mL), and K2I (11.02 µg/mL) being the most efficient producers.

#### 3.2.3 Hydrogen cyanide (HCN) production

PGPR can suppress phytopathogens through several mechanisms, including HCN production. In the present study, all potential isolates were screened for HCN activity (Figure 1C; Table 2).

Among rhizospheric isolates, MC3, SR3 showed strong HCN production, while MC4 and MC6 exhibited weak activity. Whereas, K1III showed weak activity and other isolates displayed no detectable activity (Figure 1C; Table 2).

Overall, only a few rhizospheric isolates produced HCN, with stronger activity observed in MC3 and SR3. In contrast, endophytic isolates showed limited activity, suggesting that rhizospheric bacteria are generally more proficient at HCN production compared to endophytes.

#### 3.2.4 Siderophore production

**FIGURE 3:**
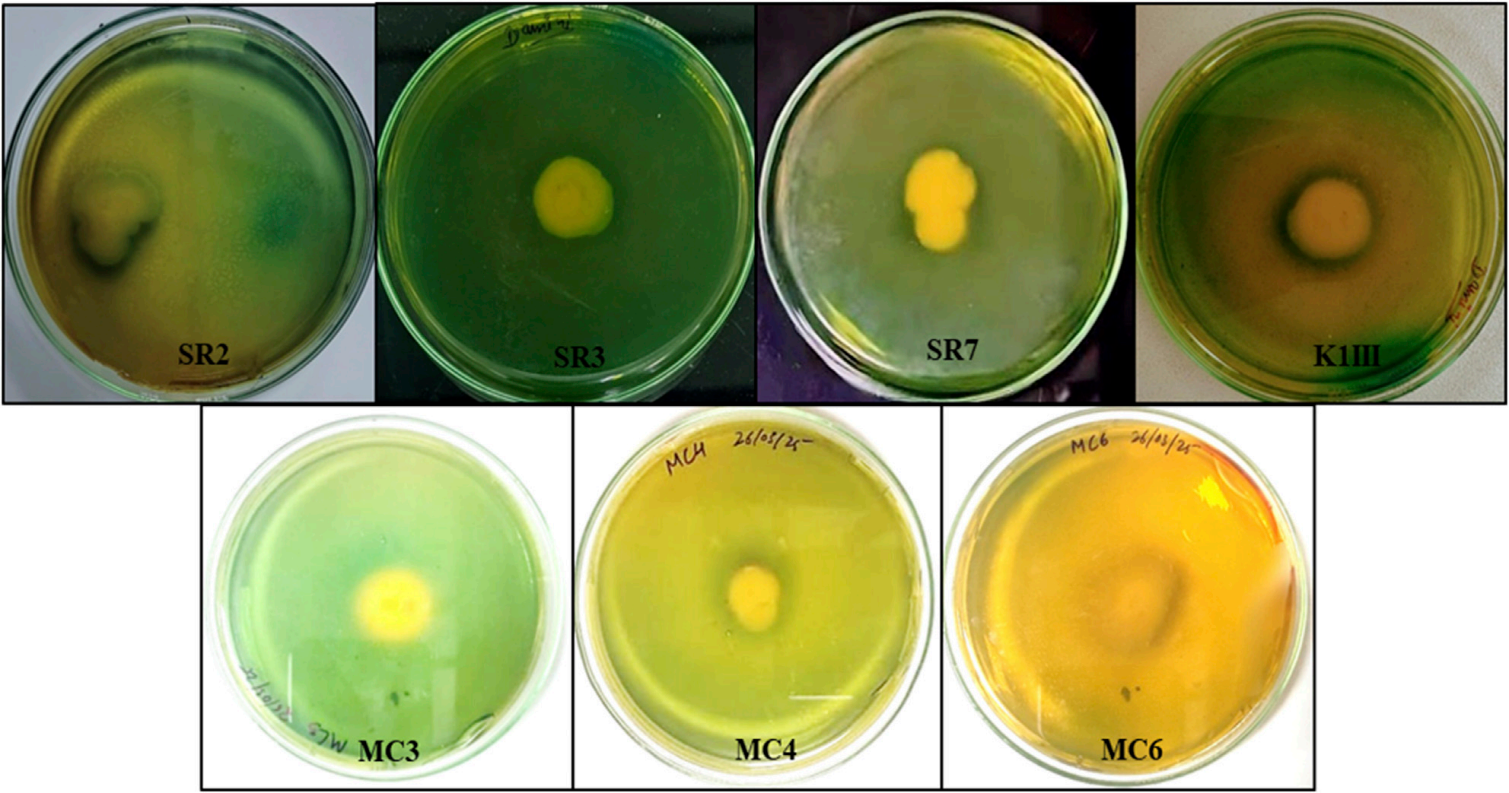
Orange halo formation around bacterial isolates indicated siderophore production on CAS agar.

The chrome azurol S (CAS) assay was used to detect siderophore production. In this assay, siderophores act as iron scavengers by interacting with the Fe-CAS-hexadecyltrimethylammonium bromide complex, resulting in a color change from blue to orange, which indicates their presence (Table 2; Figure 3).

Among rhizospheric isolates, MC1, MC3, MC4, MC6, SR2, SR3, and SR7 exhibited siderophore activity, with MC6 followed by MC3 showing strong production (++). In contrast, MC8, MC9, SR4, and SR9 showed no activity. For endophytic isolates, K1III and K2III demonstrated siderophore production, while others were negative (Table 2).

Overall, siderophore activity was more prominent among rhizospheric isolates compared to endophytes, suggesting their stronger potential in iron acquisition and pathogen suppression.

#### 3.2.5 Ammonia production

Ammonia production is an important PGPR trait, providing a readily available nitrogen source that supports root and shoot elongation and overall plant biomass. In the present study, all isolates were screened for ammonia production (Table 2; Figure 1D; Supplementary Figure S4).

Among rhizospheric isolates, SR2 and SR3 showed strong activity (++), while MC3, MC4, and MC6 exhibited weak activity (+). Most other rhizospheric isolates were negative. For endophytic isolates, K1III and K2III displayed weak activity (+), whereas the rest showed no detectable production.

Overall, two rhizospheric isolates demonstrated strong ammonia production, while two endophytic isolates exhibited weak activity, indicating that rhizospheric bacteria are generally more proficient in ammonia production compared to endophytes.

#### 3.2.6 Biofilm production

Biofilm formation is a survival strategy that provides protection against environmental stress, improves nutrient access, and enhances resistance to antibiotics and host defenses. In the present study, all isolates were screened for biofilm production (Figure 1E; Table 2; Supplementary Figure S5).

Among rhizospheric isolates, SR2 and SR4 exhibited strong biofilm production (++), while SR3 and MC3 showed weak activity (+). Most other rhizospheric isolates were negative. In the case of endophytes, K1III, K1IV, and K2III displayed weak biofilm production (+), whereas the rest showed no activity.

Overall, two rhizospheric isolates demonstrated strong biofilm production, while three endophytic isolates exhibited weak activity. This indicates that rhizospheric bacteria are generally more efficient in biofilm formation compared to endophytes.

#### 3.2.7 EPS production

EPS facilitate bacterial adhesion, biofilm formation, and protection under stress conditions. In the present study, all isolates were screened for EPS production (Figure 1F; Table 2; Supplementary Figure S6).

Among rhizospheric isolates, MC3, MC4 and SR3 showed weak EPS production (+), while SR2 and SR4 exhibited strong activity (++). In contrast, most other rhizospheric isolates were negative. For endophytic isolates, K1III, K1IV, and K2III displayed weak EPS production (+), whereas the rest showed no activity.

Overall, two rhizospheric isolates demonstrated strong EPS production, while three endophytic isolates exhibited weak activity. This indicates that rhizospheric bacteria are generally more proficient in EPS production compared to endophytes.

#### 3.2.8 Catalase activity

Catalase activity is a vital trait that enables bacteria to detoxify hydrogen peroxide, thereby enhancing survival under oxidative stress and supporting plant colonization. In the present study, potential isolates were screened for catalase activity (Supplementary Figure S7; Table 2).

Among rhizospheric isolates, MC1, MC3, MC4, MC6, MC8, MC9, SR2, SR3, SR4, SR7, and SR9 all demonstrated positive activity. Similarly, endophytic isolates K1III, K1IV, K2I, and K2III also showed catalase activity. No negative results were observed, indicating that catalase production is a common trait among both rhizospheric and endophytic bacteria.

Overall, the universal presence of catalase activity across isolates suggests strong adaptive potential and highlights their functional role in plant-microbe interactions.

#### 3.2.9 Biocontrol activity of efficient rhizospheric and endophytic bacterial isolates against fungal plant pathogens

**FIGURE 4:**
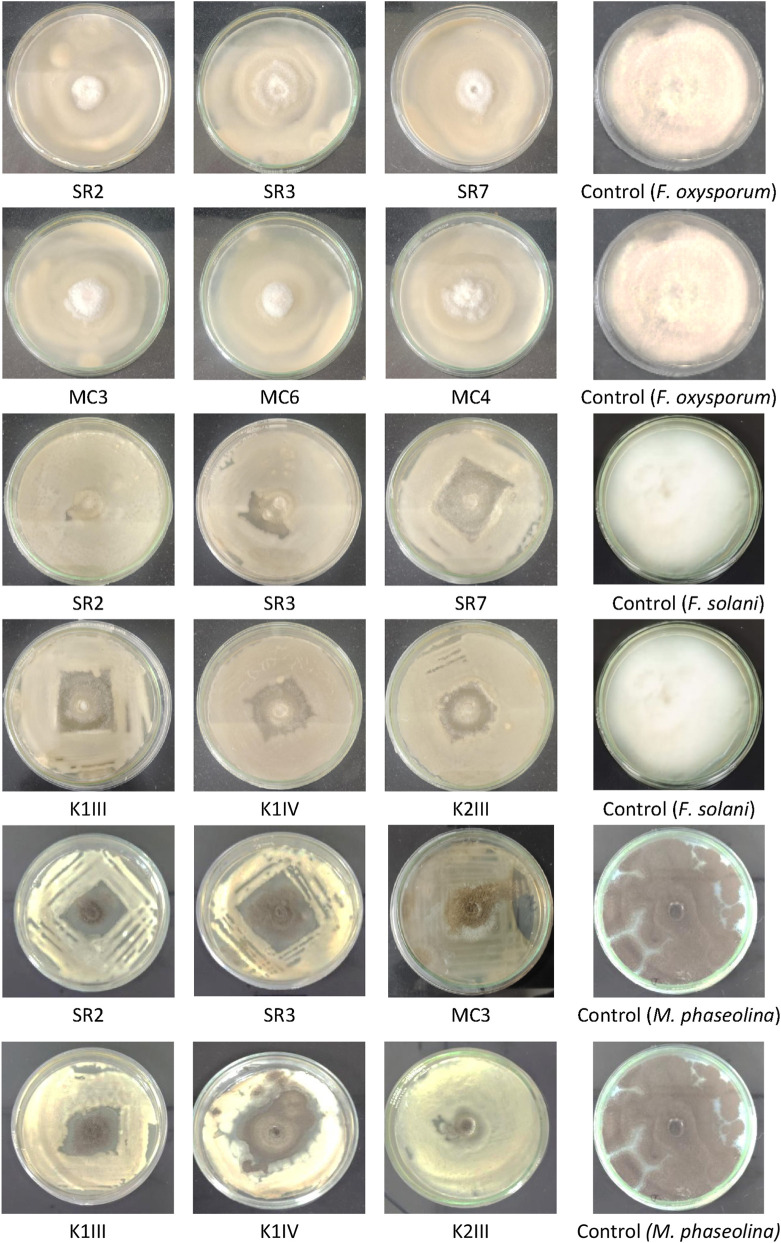
*In vitro* screening of endophytic and rhizospheric bacteria for biocontrol activity using the dual culture assay against *F. oxysporum*, *F. solani* and *M. phaseolina.* Representative photographs are shown. Original unprocessed images corresponding to this figure are provided in Supplementary material S2.

**FIGURE 5:**
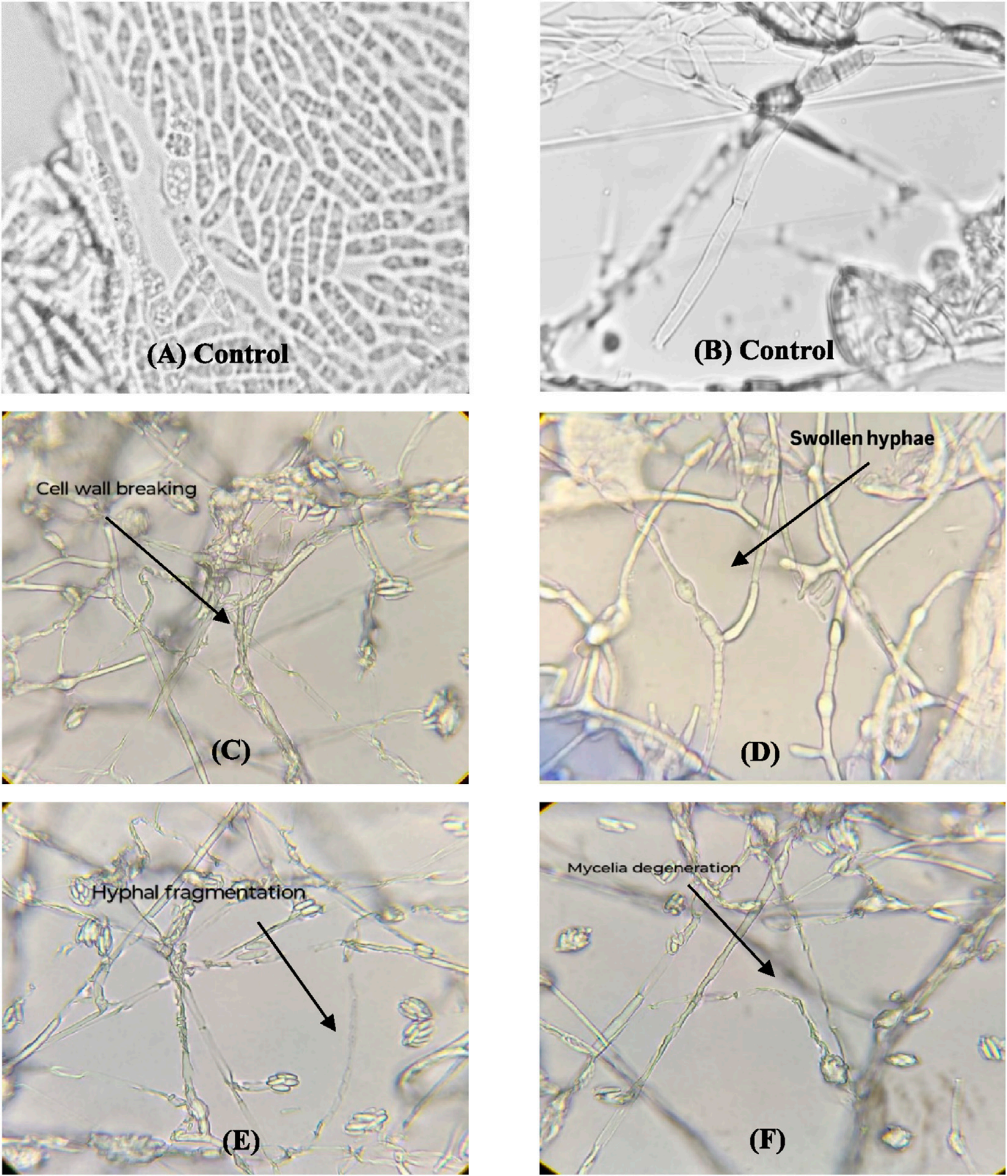
Microscopic differences between fungal hyphae in control and fungal hyphae bio-controlled by plant growth promoting isolates. **(A,B)** microscopic views of untreated fungal hyphae (Control). **(C–F)** showed deformities in mycelia of treated fungi such as cell wall breaking, swollen hyphae, hyphal fragmentation and mycelia degeneration, *etc.* Images were acquired at 400x magnification.

Potential phosphate-solubilizing isolates were evaluated for their *in vitro* antagonistic potential against major fungal pathogens (*F. oxysporum*, *F. solani* and *M*. *phaseolina*) (Figure 4). Several rhizospheric isolates (MC3, MC4, MC6, SR2, SR3, SR7) and endophytic isolates (K1III, K1IV, K2III) demonstrated strong biocontrol activity, showing fungal growth inhibition and deformities in fungal hyphae such as fragmentation, swelling and degeneration (Figures 5A–F). Individual images of the dual culture assays shown in Figure 4 were combined only for presentation purposes and no selective enhancement or manipulation affecting the scientific interpretation of the results was performed. The original unprocessed images corresponding to Figure 4 have been provided as supplementary Data S2.

##### 3.2.9.1 PGI of potential bacterial isolates against fungal pathogens

Biocontrol assays revealed variable inhibition across isolates (Table 3). In the case of *F*. *oxysporum*, the isolates SR2 and MC3 demonstrated the strongest inhibitory activity, with inhibition rates of 86.2% and 80.2%, respectively. These were followed by SR7 (78.2%) and K1IV (70.3%), which also showed considerable suppression of pathogen growth.

**TABLE 3:** PGI of potential bacterial isolates against fungal pathogens.

Isolate	*F. oxysporum*	*F. solani*	*M. phaseolina*
MC3	80.2%^b^	75.1%^a^	57.5%^b^
MC4	55.1%^e^	68.8%^b^	–
MC6	25%^g^	45.6%^f^	60.6%^a^
SR2	86.2%^a^	66.8%^c^	50.1%^d^
SR3	–	–	46.6%^e^
SR7	78.2%^c^	57.9%^d^	–
K1III	52.70%^f^	46.7%^e^	45.70^f^
K1IV	70.3%^d^	–	–
K2III	–	68.4%^b^	54.7%^c^

Means with different lowercase letters in the same column differ significantly atp<0.05, according to Tukey’s *post hoc* test.

Similarly, against *F*. *solani*, MC3 (75.1%), K2III (68.4%) and MC4 (68.8%) were the most effective, while SR2 (66.8%), SR7 (57.9%) and K1III (46.7%) exhibited notable inhibition, though at comparatively lower levels.

In the case of *M*. *phaseolina*, MC6 (60.6%), MC3 (57.5%) and K2III (54.7%) displayed the highest inhibitory potential. SR2 (50.1%), SR3 (46.6%) and K1III (45.70%) also contributed to pathogen suppression, though their activity was moderate compared to the leading isolates.

Generally, bacterial isolates MC3, SR2 and K1III exhibited multifarious PGP characteristics and were subsequently selected for evaluation under abiotic stress-tolerance assays.

### 3.3 Abiotic stress-tolerance activity of potential bacterial isolates

**FIGURE 6:**
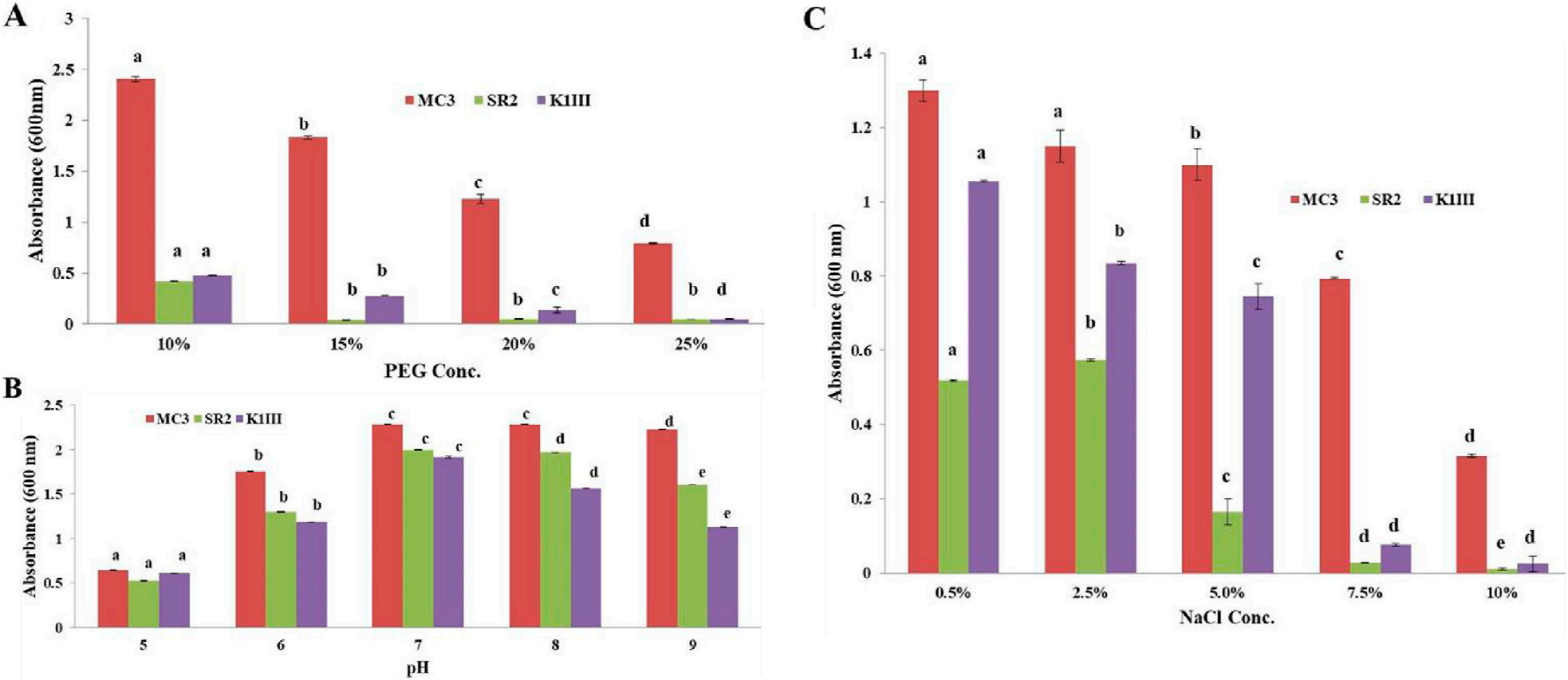
Abiotic stress-tolerance activities of potential isolates **(A)** Drought stress (PEG10% to 25%) **(B)** pH tolerance (5–9) **(C)** Salinity stress (NaCl 0.5% to 10%). Values are expressed as mean ± SD. Means with different lowercase letters in the same color bars differ significantly at p < 0.05, according to Tukey’s *post hoc* test.

Abiotic stresses such as drought, salinity, and extreme pH strongly influence microbial survival and plant productivity. In this study, potential isolates MC3, SR2, and K1III were evaluated for tolerance to various abiotic conditions (Figures 6A–C).

MC3 and K1III showed strong resistance to salinity up to 5% NaCl. However, MC3 was able to survive even under high salt concentrations, i.e. 10%, confirming its superior salt tolerance (Figure 6C). MC3 was the most tolerant under pH stress, retaining stable activity in neutral to alkaline ranges (pH 7–9). SR2 was moderately tolerant, growing best at pH 7–8. K1III was the least tolerant, and its activity dropped sharply after pH 7 (Figure 6B).

Under PEG-induced osmotic stress, MC3 once again showed better tolerance, keeping high activity at 10% (2.40) and moderate activity up to 25% (0.79). K1III had medium tolerance, with activity slowly decreasing as concentrations increased. On the other hand, SR2 was very sensitive, showing little activity even at 10% (0.42) and almost complete inhibition at higher PEG levels (6A).

Overall, MC3 was found to be the most resilient isolate, able to cope with many abiotic stresses. It sustained activity even when the salinity was low, the pH was neutral to alkaline, and PEG-induced osmotic stress. This highlights that it could improve plant growth under adverse environments.

### 3.4 Effect of different PGP treatments on chickpea (*Cicer arietinum*) plant growth

**FIGURE 7:**
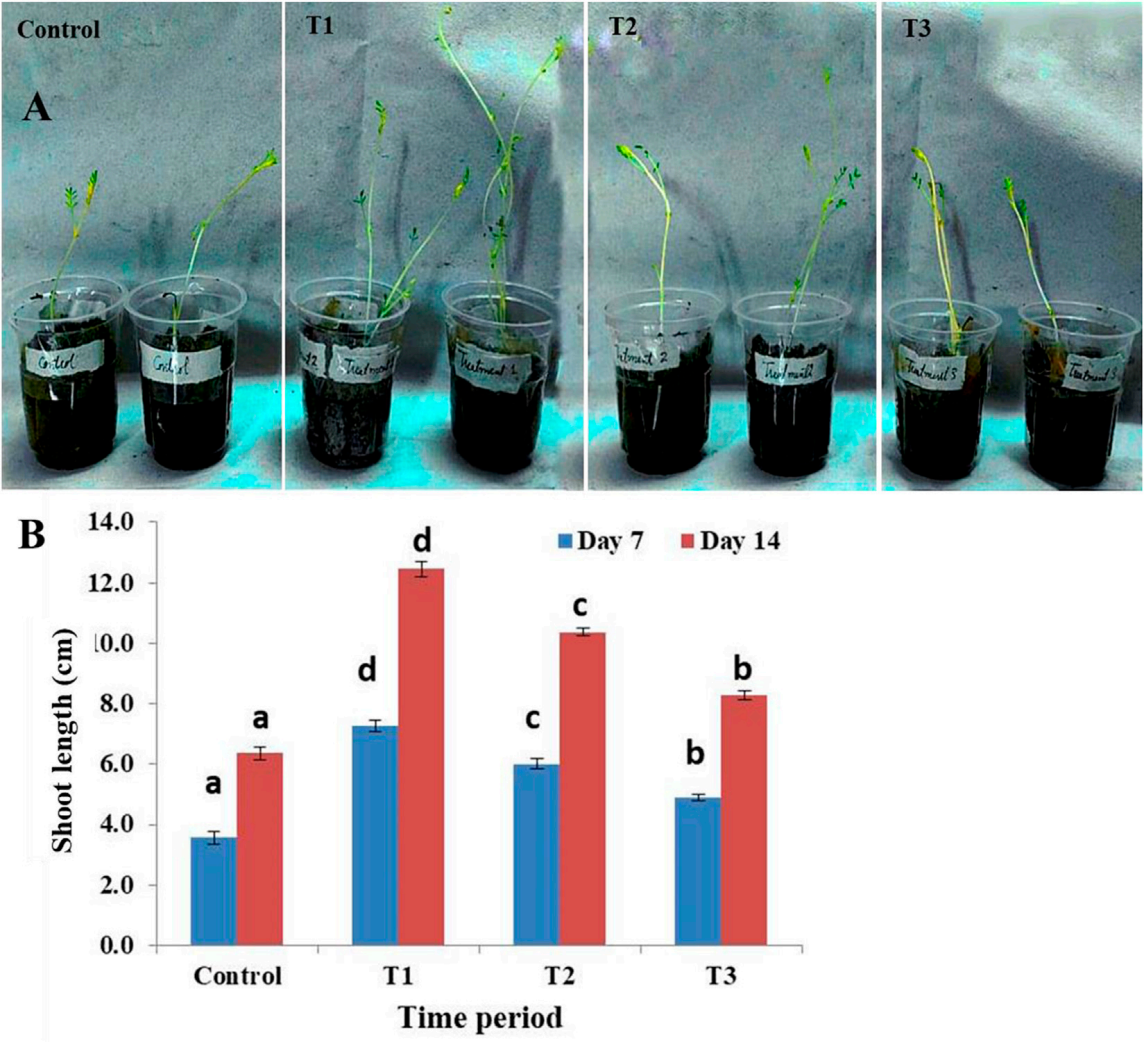
Effect of selected PGP bacterial isolates on the growth of chickpea; Control (uninoculated plants), T1 (MC3), T2 (SR2) and T3 (K1III). **(A)** Effect of selected bacterial treatments on growth parameters of chickpea plants **(B)** Effect of bacterial treatments on shoot length of chickpea plants at different growth intervals. Values are expressed as mean ± SD. Means with different lowercase letters in the same color bars at Day 7 (blue) and Day 14 (red) differ significantly at p < 0.05, according to Tukey’s *post hoc* test.

To evaluate the efficacy of bioformulation treatments, four experimental groups were established: (i) the control group, (ii) Treatment 1 (T1) with inoculation of MC3, (iii) Treatment 2 (T2) with inoculation of SR2 and (iv) Treatment 3 (T3) with inoculation of K1III. The impact of these treatments on early chickpea growth was assessed at 7-day intervals after seed sowing (Figures 7A,B). All inoculated treatments significantly enhanced plant growth compared to the uninoculated control (Figures 7A,B).

### 3.5 Biological and molecular characterization of potential bacterial isolates

On the basis of multifarious PGP characterstics**,** the promising bacterial isolates MC3, MC4, MC6, SR2, SR3, K1III and K2III were subsequently selected for detailed morphological and biochemical characterization.

#### 3.5.1 Morphological and gram staining characteristics

Morphological observations revealed distinct colony features among selected isolates. Gram staining and colony traits such as size, shape, color, and surface texture are summarized in Table 4 **.** Microscopic observations confirmed rod-shaped morphology across all isolates (Table 4).

**TABLE 4:** Gram staining, colony morphology and biochemical characterization of potential endophytic as well as rhizospheric isolates.

Morphological characterstics	MC3	MC4	MC6	SR2	SR3	K1III	K2III
Gram staining	Gram (−)ve	Gram (+)ve	Gram (−)ve	Gram (+)ve	Gram (−)ve	Gram (−)ve	Gram (+)ve
Shape	Rods	Rods	Rods	Rods	Rods	Rods	Rods
Colony size/shape	Small circular	Medium circular	Medium circular	Medium irregular	Medium irregular	Small circular	Small irregular
Colony color	Yellowish white	Yellow	White	Creamy white	Creamy white	White	Creamy white
Surface	Smooth	Smooth	Smooth	Smooth	Smooth	Smooth	Smooth

Biochemical test	MC3	MC4	MC6	SR2	SR3	K1III	K2III
Glucose fermentation	A	A	AG	A	AG	–	A
Sucrose fermentation	A	A	AG	A	AG	–	A
Lactose fermentation	A	–	AG	–	AG	–	–
Mannitol fermentation	A	A	AG	A	AG	A	A
Nitrate reduction	+	–	+	–	–	–	–
Triple sugar iron test	+	+	+	–	–	–	–
Catalase	+	+	+	+	+	+	+
Methyl red	_	+	–	+	–	–	+
Indole	–	–	+	–	–	–	–
Voges–Proskauer	+	–	+	–	+	–	–
Citrate utilization	+	+	+	+	+	+	+
Oxidase	–	–	–	–	–	+	–
Urease utilization	–	–	+	–	+	–	–
Motility	+	+	–	+	+	+	+

- negative, + positive, A-acid production, AG-acid and gas production.

#### 3.5.2 Biochemical characterization

Biochemical assays revealed diverse metabolic capabilities (Table 4). Rhizospheric isolates MC3, MC4, and MC6 showed positive results for sugar fermentation (glucose, sucrose and mannitol), catalase activity and citrate utilization, with variations in nitrate reduction, methyl red and indole tests (Table 4). Endophytic isolates K1III and K2III, along with rhizosheric isolates SR2 and SR3, displayed positive activity for citrate, catalase, motility, and mannitol sugar fermentation, with SR3 showing gas production in multiple sugars (Table 4).

Based on the results of colony morphology, Gram staining and biochemical profiles, the preliminary identification of potential isolates are listed in Table 5.

**TABLE 5:** Preliminary identification of potential isolates based on colony morphology, Gram staining and biochemical characterstics.

S. No.	Isolate code	Name of bacterial isolates
1	MC3	*Lelliottia* sp
2	MC4	*Bacillus* sp
3	MC6	*Klebsiella* sp
4	SR2	*Bacillus* sp
5	SR3	*Enterobacter* sp
6	K1III	*Pseudomonas* sp
7	K2III	*Bacillus* sp

#### 3.5.3 Molecular identification of most promising bacterial isolate MC3

PCR amplification of the 16S rRNA gene from isolate MC3 produced a distinct amplicon of approximately 1,500 bp, as visualized on 1.2% agarose gel stained with ethidium bromide (Supplementary Figure S8), confirming successful amplification of the target gene. Subsequent BLASTn analysis of the 16S rRNA gene sequence confirmed isolate MC3 was identified as *L*. *aquatilis,* exhibiting 97.93% sequence similarity with its closest taxonomic relative and indicating a strong genetic relationship (data S1, Table 6).

**TABLE 6:** Taxonomic identification of *L*. *aquatilis* (MC3) based on 16SrRNA sequence.

Taxonomic rank	Classification
Kingdom	Pseudomonadati
Phylum	Pseudomonadota
Class	Gammaproteobacteria
Order	Enterobacterales
Family	Enterobacteriaceae
Genus	*Lelliottia*
Species	*Lelliottia aquatilis*

##### 3.5.3.1 Phylogenetic tree construction

**FIGURE 8:**
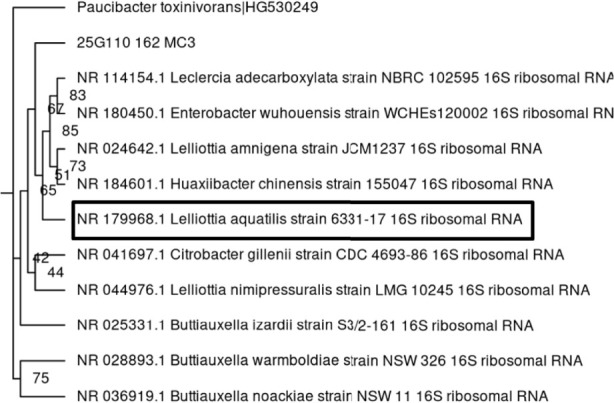
Phylogenetic tree of *L*. *aquatilis* based on the 16S rRNA sequence.

Phylogenetic analysis using Maximum Likelihood (IQ-TREE, Kimura model, 1,000 bootstraps) placed MC3 within *Enterobacterales*, closely related to *L*. *aquatilis* (Figure 8).

Overall, the findings indicate that the tested isolates had multiple PGPR traits but differed in their efficiency in different PGPR assays. In particular, MC3 emerged as a superior isolate due to its high phosphate solubilization capability, IAA and siderophore production, ammonia release, catalase activity, and promising biocontrol activity against *F. oxysporum*, *F. solani* and *M. phaseolina*. MC3 showed greater tolerance to abiotic stress factors and has a positive influence on chickpea growth. For this reason, MC3 was chosen for molecular identification, which revealed its identity as *L*. *aquatilis*. The sequence was submitted in GenBank under the accession number PZ232191. These findings highlight MC3 as the most promising candidate for further development as a bioinoculant in sustainable agriculture.

## 4 Discussion

PGPR are increasingly recognized as eco-friendly alternatives to chemical fertilizers and pesticides because of their ability to enhance nutrient availability, stimulate plant growth, and suppress pathogens (Ali et al., 2026; Pathak et al., 2026). The present study demonstrated that rhizospheric and endophytic bacterial isolates exhibit a wide range of PGP traits, including phosphate solubilization, IAA and siderophore production, ammonia release, catalase activity, EPS secretion, biofilm formation, biocontrol potential, and tolerance to abiotic stresses. Recent studies have revealed that effective PGPR strains should possess several complementary mechanisms instead of only one plant-beneficial trait, thus ensuring more efficient functioning when applied in the field conditions (Andrade et al., 2023; Wahab et al., 2024).

These traits are central to PGPR-mediated improvements in nutrient availability, root development, and plant resilience under stress (Egamberdieva et al., 2017; Sharma et al., 2020). Additionally, the results revealed considerable variability among screened isolates, with rhizospheric strains generally outperforming endophytes.

Among the isolates examined, SR4, SR7, K1III, MC4, MC6 and SR2 demonstrated enhanced phosphate-solubilizing capacity, as evidenced by their higher soluble phosphorus concentrations in broth culture. Notably, although MC3, SR3, K1III, SR7, K1IV and SR2 showed larger halo zones in qualitative screening on PVK agar, quantitative analysis showed that SR4 and SR7 released higher amounts of soluble phosphorus in broth culture. Such differences between solid and liquid-based assays have been reported earlier and are generally attributed to the differences in diffusion of organic acids, composition of the medium and metabolic activity of microbes under different growth conditions (Baig et al., 2010; Blanco-Vargas et al., 2020). These results indicate that qualitative and quantitative assays determine different aspects of phosphate solubilizing efficiency and should be interpreted in a complementary manner. Phosphate solubilization is a critical mechanism for mobilizing insoluble phosphate into plant-available forms, and has been widely reported in PGPR such as *Pseudomonas* and *Bacillus* (Andrade et al., 2023; Ali et al., 2026).

Likewise, IAA production was reliably detected in all the screened isolates. Earlier research has shown that PGPR producing IAA increase nutrient uptake, facilitate root growth and lateral root formation and biomass production in crops (Ayele et al., 2021).

Siderophore production was another important trait, isolates MC1, MC3, MC4, MC6, SR2, SR3, SR7, K1III and K2III found positive for siderophore production. Siderophores bind iron, restraining pathogen activity and enhancing plant nutrition (Compant et al., 2019; Lotfi et al., 2022). Ammonia production by MC3, MC4, MC6, SR2, SR3, K1III and K2III further enhances plant nutrition, in line with previous results that PGPR producing ammonia plays a role in nitrogen cycling (Ali et al., 2026).

Catalase production was widespread, with all strains capable of breaking down hydrogen peroxide and resisting oxidative stress (Falcon-Pineiro et al., 2026). Studies by Compant et al. (2019) revealed that catalase-positive PGPR are more suited to rhizosphere conditions, this enzymatic resilience is essential for colonization in aerobic environments. Research investigations that link EPS production to enhanced drought resilience and pathogen suppression are corroborated by the fact that EPS and biofilm formation, which are especially strong in SR2 and SR4, further improve root colonization and stress-tolerance (Haque et al., 2020; Devi et al., 2023).

*F. oxysporum*, *F. solani* and *M. phaseolina* were inhibited by MC3, SR2, MC6, MC4, SR3, SR7, K1III, K1IV, and K2III, according to biocontrol assays. Similar to previous reports of PGPR-mediated suppression of soil-borne pathogens through antifungal metabolites, siderophore release, and competition for nutrients, MC3 demonstrated exceptionally strong inhibition (80.2% against *F. oxysporum*, 75.1% against *F. solani*). Hyphal abnormalities that are characteristic of PGPR antagonism, such as fragmentation, swelling, and degeneration, were verified by microscopic observations (Hu et al., 2025; Pallavi et al., 2023).

Abiotic stress tests demonstrated MC3’s resistance to drought (PEG 25%), salinity (up to 5% NaCl and survival at 10% NaCl), and pH extremes (5–9). This is essential for PGPR to survive in arid and stressed environments, and has been previously reported in salt-tolerant PGPRs that enhance plant growth through changes in phytohormone, antioxidant and osmolyte content (Egamberdieva et al., 2017). In the present study, the ability of the isolates to tolerate salinity up to 5% NaCl, coupled with survival at 10% NaCl is indicative of strong osmotolerance and suggests that the isolates selected may remain functional under saline and stress-prone agricultural conditions. Therefore, these isolates could be potential microbial inoculants for sustainable crop production in salt-affected soils (Haque et al., 2020; Pallavi et al., 2023; Wahab et al., 2024). Multistress tolerance of MC3 highlights its potential use in a range of agro-climatic conditions, including the Himalayan region, which is known for high levels of abiotic stresses.

PGP traits were functionally validated through chickpea growth assays, where MC3 produced the highest enhancement in shoot length. Based on the above multifaceted results, MC3 was chosen for molecular assessment. This was based on its consistent expression of almost all PGPR traits, best biocontrol potential and superior tolerance to abiotic stress, making it the most efficient and reliable isolate. Latest research have indicated that successful PGPR candidates possess multiple complementary mechanisms that work together to enhance nutrient acquisition, stimulate plant growth, increase stress tolerance, and suppress phytopathogens (Andrade et al., 2023; Wahab et al., 2024). Thus, the multifunctionality of MC3 highlights its potential as a promising candidate for a bioinoculant.

16S rRNA sequencing identified MC3 as *L*. *aquatilis* of the order Enterobacterales. Although *Lelliottia* species have been described in aquatic habitats (Kampfer et al., 2018), their role as PGPR is less documented, making this finding novel. The identification of MC3 as *L*. *aquatilis* strengthens the diversity of bacterial taxa currently classified as PGPR. The majority of commercial biofertilizers are primarily comprised of genera of *Bacillus*, *Pseudomonas*, *Azospirillum* and *Rhizobium* (Andrade et al., 2023; Tariq et al., 2025). Among these, *Bacillus subtilis* and *Pseudomonas fluorescens* are considered as model PGPR due to their efficient root colonization, mobilization of nutrients, production of plant hormones and their ability to suppress pathogens (Wahab et al., 2024; Giri and Virk, 2025). Interestingly, *L*. *aquatilis* MC3 showed several characteristics similar to these known PGPR strains including phosphate solubilization, IAA production, siderophore production, biofilm formation, broad-spectrum antifungal activity and abiotic stress-tolerance (Joshi et al., 2023; Wahab et al., 2024). These results indicate that MC3 has multifunctional characteristics competitive with several commercially exploited PGPR strains and could be a valuable addition to the pool of beneficial agricultural microorganisms.

The ecological importance of such finding is especially pertinent in the Himalayan region. The Himalayan region is one of the world’s most diverse and least explored reservoirs of microbial biodiversity, characterized by extreme environmental heterogeneity, fluctuating temperatures, nutrient-poor soils, steep altitudinal gradients and diverse plant communities. The environmental constraints impose high selective pressures leading to the evolution of metabolically versatile, stress-tolerant, and functionally diverse microorganisms capable of surviving in challenging ecological conditions (Compant et al., 2019; Andrade et al., 2023; Falcon-Pineiro et al., 2026). Recent studies have shown that microorganisms from mountain ecosystems generally possess unique adaptive traits such as efficient nutrient mobilization, phytohormone production, exopolysaccharide synthesis, pathogen suppression, and drought, salinity, and temperature tolerance, making them potential resources for sustainable agriculture and climate-change adaptation (Joshi et al., 2023; Wahab et al., 2024; Giri and Virk, 2025).

The recognition of MC3 as a member of the Enterobacteriaceae family, however, requires a thorough discussion of biosafety issues prior to large-scale agricultural use. Several members of Enterobacteriaceae, such as plant-associated *Enterobacter*, *Kosakonia*, *Klebsiella* and related genera have been extensively documented as efficacious PGPR and studied for biofertilizer and bioinoculant development (Compant et al., 2019; Da Silva et al., 2024; Falcon-Pineiro et al., 2026). It has been demonstrated by several studies that the rhizospheric and endophytic strains of these genera enhance plant growth significantly by biological nitrogen fixation, phosphate solubilization, phytohormone production, nutrient acquisition and stress mitigation (Andrade et al., 2023; Wahab et al., 2024). Additionally, patented bioinoculant technologies based on members of Enterobacteriaceae, including *Klebsiella*-based formulations, have proved the agricultural relevance and commercial potential of this group of bacteria (Triplett et al., 2008). Ecological origin strongly influences microbial functionality with strains from plant rhizospheres and endophytic niches often adapted to beneficial plant-associated lifestyles that are markedly different from their clinical counterparts. Recent advances in plant microbiome research further suggest that rhizospheric and endophytic habitats act as selective environments that shape microbial diversity and enrich microorganisms with beneficial traits associated with plant growth promotion, stress resilience, and disease suppression (Mitter et al., 2019; Compant et al., 2019; Joshi et al., 2023; Ali and Moon, 2025). Thus, the multifunctional characteristics exhibited by *L*. *aquatilis* MC3 may be an adaptation to its plant-associated ecological niche and support its potential as a candidate bioinoculant for sustainable agriculture.

However, taxonomic affiliation solely is inadequate in order to establish biosafety. Some members of Enterobacteriaceae are known opportunistic human pathogens, and thus thorough biosafety assessment is still required before recommending *L*. *aquatilis* MC3 as a commercial bioinoculant. While strains isolated from rhizospheric and endophytic environments are usually considered to be of low risk to the environment, as they are derived from natural plant-associated ecosystems (Mitter et al., 2019; Compant et al., 2019; Joshi et al., 2023; Malusa and Vassilev, 2014). Current recommendations for the development of microbial inoculants require a rigorous evaluation of strains at the strain rather than the taxonomic level (Compant et al., 2019; Mitter et al., 2019; Wahab et al., 2024). Thus, additional research on whole-genome sequencing, screening of virulence-associated genes, profiling of antimicrobial resistance, assessment of pathogenicity, and environmental risk analysis should be conducted for safe application of MC3 in agriculture. The present study has also some limitations. The experiments were mainly carried out under laboratory and pot culture conditions, and the performance of MC3 under field conditions remains to be validated. Moreover, abiotic stress-tolerance was tested through *in vitro* bacterial assays and not directly tested for plant stress alleviation under drought, salinity and other environmental stresses. The molecular characterization was limited to 16S rRNA gene sequencing and therefore the genetic basis of the observed PGP and biocontrol traits is unexplored. Future studies should focus on field validation, formulation development, whole genome sequencing, genome-based functional characterization, biosafety assessment and evaluation of plant performance under natural stress conditions to establish practical applicability of *L*. *aquatilis* MC3 as a bioinoculant.

Together, the results from the present study show a unique combination of PGP, biocontrol and abiotic stress-tolerance traits in *L*. *aquatilis* MC3. These findings broaden the existing knowledge of PGPR diversity, underline the ecological value of Himalayan microbial resources and identify *L*. *aquatilis* as a potential candidate for future biofertilizer and biocontrol development for sustainable and climate-resilient agriculture.

## 5 Conclusion

The multifunctionality of rhizospheric and endophytic bacterial isolates obtained from *M*. *esculenta* and termite mound–associated plants was demonstrated in this study. The isolates displayed several PGPR traits including phosphate solubilization, production of IAA, production of siderophore, ammonium, biofilm, EPS, and catalase activity. Among them, isolate MC3 was found to be superior to all the other isolates in terms of showing strong antagonism against fungal pathogens, tolerance to abiotic stress conditions and substantial enhancement of plant growth.

MC3 was identified as *L*. *aquatilis*, an Enterobacterales, bringing novelty to the study by revealing a previously understudied group of PGPRs. Its diversity of traits, effective PGP, biocontrol and stress-tolerance make MC3 a potential candidate for biofertilizer and biocontrol formulation. Our results are consistent with a global shift towards less chemical use and more sustainable farming. The future step in research needs to incorporate field-trials, formulation of microbial consortia and genomics for accelerating the transfer to climate-resilient, food secure systems.

## Data Availability

All data generated or analyzed during this study are included in the published article and its supplementary information file. The 16S rRNA gene sequence of *L*. *aquatilis* strain MC3 generated during this study has been deposited in the NCBI GenBank database under accession number PZ232191 at https://www.ncbi.nlm.nih.gov/nuccore/PZ232191. In addition, the complete FASTA sequence has been provided as Supplementary material S1.

## Glossary

ANOVA: Analysis of Variance
BLAST: Basic Local Alignment Search Tool
CAS: Chrome Azurol S
CFU: Colony-Forming Unit
CMC: Carboxymethyl Cellulose
DNA: Deoxyribonucleic Acid
EPS: Exopolysaccharide
HCN: Hydrogen Cyanide
IAA: Indole-3-Acetic Acid
MEGA: Molecular Evolutionary Genetics Analysis
NaCl: Sodium Chloride
NCBI: National Center for Biotechnology Information
OD: Optical Density
PCR: Polymerase Chain Reaction
PDA: Potato Dextrose Agar
PEG: Polyethylene Glycol
PGI: Percentage Growth Inhibition
PGP: Plant Growth-Promoting
PGPR: Plant Growth-Promoting Rhizobacteria
PSI: Phosphate Solubilization Index
PVK: Pikovskaya’s Medium
SD: Standard Deviation
TCP: Tricalcium Phosphate
TSB: Tryptic Soy Broth
16S rRNA: 16S Ribosomal Ribonucleic Acid
F. oxysporum: Fusarium oxysporum
F. solani: Fusarium solani
M. phaseolina: Macrophomina phaseolina
P. pashia: Pyrus pashia
C. carandas: Carissa carandas
L. aquatilis: Lelliottia aquatilis
