# Supplementary material for: Integrative functional profiling of rhizospheric and endophytic bacteria reveals Lelliottia aquatilis as a multifunctional PGPR in Himalayan ecosystems
Source: Front Syst Biol. 2026 Aug 3;6:1872892. doi: 10.3389/fsysb.2026.1872892 (PMC13477884; doi:10.3389/fsysb.2026.1872892)
Supplement: Supplementary file 1 [file DataSheet1.docx]

**Integrative Functional Profiling of Rhizospheric and Endophytic Bacteria Reveals *Lelliottia aquatilis* as a Multifunctional PGPR in Himalayan Ecosystems**

Shikha Devi^1^, Riya Chandel^3^, Damini Thakur^1^, Shweta Pathania^1^, Simran Rana^1^, Simran^1^, Nishtha Pathak^1^, Poonam Katoch^1^, Taniya Tyagi, Sushila Devi^1^, Arvind Kumar Bhatt^2^, Sveta Thakur^3^*

^1^Department of Microbiology, Himachal Pradesh University Shimla, 171005, India

^2^Department of Biotechnology, Himachal Pradesh University Shimla, 171005, India

^3^Department of Biosciences, Himachal Pradesh University Shimla, 171005, India

*****Correspondence: [svetathakur19@gmail.com](mailto:svetathakur19@gmail.com)

**Supplementary Figure 1: Soil sampling from *Myrica esculenta* rhizosphere and termite mound-associated plants.**

**Supplementary Figure 2:Trend of pH during phosphate solubilization by potential isolates (a) rhizospheric and (b) endophytic bacteria.**

**Supplementary Figure 3: IAA production by potential bacterial isolates.**

**Supplementary Figure 4: Tubes showing ammonia production by potential bacterial isolates.**

**Supplementary Figure 5: Biofilm production by potential bacterial isolates.**

**Supplementary Figure 6: Tubes showing EPS production indicated by white precipitate.**

**Supplementary Figure 7: Catalase production by potential bacterial isolates.**

**Supplementary Figure 8: Agarose gel electrophoresis of PCR products.**

**Supplementary Figure 9. TIF image of Fig. 4 (*In vitro* screening of endophytic and rhizospheric bacteria for biocontrol activity using the dual culture assay against *F. oxysporum*, *F. solani* and *M. phaseolina).***

**Supplementary Table S1. the geographical coordinates and details of the collected samples.**

**Supplementary data 1. 16S rDNA consensus sequence**

**Supplementary data 2. Representative images of the dual culture assay for biocontrol activity by potential bacterial isolates.**

**Fig. S1: Soil sampling from *Myrica esculenta* rhizosphere and termite mound associated plants.**

pH

| Incubation period | MC3 | MC4 | MC6 | SR2 | SR3 | SR4 | SR7 |
| --- | --- | --- | --- | --- | --- | --- | --- |
| Day 1 | 6.9 | 6.5 | 6.4 | 6.67 | 6.78 | 7.01 | 6.88 |
| Day 2 | 6.5 | 6.3 | 5.7 | 5.49 | 6.5 | 6.69 | 5.44 |
| Day 3 | 6.2 | 5.7 | 5.4 | 5.45 | 6.45 | 6.6 | 5.3 |
| Day 4 | 5.8 | 5.4 | 5.1 | 5.46 | 6.5 | 6.7 | 5.5 |
| Day 5 | 5.5 | 5.1 | 4.8 | 5.4 | 6.09 | 5.6 | 4.5 |
| Day 6 | 4.9 | 4.8 | 4.6 | 5.01 | 5.48 | 5.59 | 4.44 |
| Day 7 | 4.7 | 4.7 | 4.4 | 4.09 | 5 | 5.5 | 4.5 |

**(a)**

pH

| Incubation period | K1III | K1IV | K21 | K2III |
| --- | --- | --- | --- | --- |
| Day 1 | 7 | 7.4 | 7.67 | 7.55 |
| Day 2 | 6.8 | 6.69 | 7.7 | 7.58 |
| Day 3 | 6.78 | 6.01 | 7.79 | 6.59 |
| Day 4 | 6.77 | 6 | 7.98 | 5.55 |
| Day 5 | 5.04 | 5.53 | 6.9 | 4.5 |
| Day 6 | 5.55 | 5.3 | 6.78 | 4.08 |
| Day 7 | 5.5 | 5.4 | 5.55 | 4 |

**(b)**

**Fig. S2. Trend of pH during phosphate solubilization by potential isolates (a) rhizospheric and (b) endophytic bacteria.**

**Fig. S3. IAA production by potential bacterial isolates.**

**Fig. S4. Tubes showing ammonia production by potential bacterial isolates.**

**Control**

**K1III**

**K1IV**

**K2III**

**Fig. S5. Biofilm production by potential bacterial isolates.**

**Fig. S6. Tubes showing EPS production indicated by white precipitate.**

**Fig. S7. Catalase production by potential bacterial isolates.**

| Gel image (raw PCR product) |    |
| --- | --- |
| Gel image (purified PCR product) |    |
| Observation | A band of the product was observed at ~ 1500 bp after PCR and cleanup of the PCR product |

**Fig. S8. Agarose gel electrophoresis of PCR products.**

**Fig. S9. TIF image of Fig. 4 (*In vitro* screening of endophytic and rhizospheric bacteria for biocontrol activity using the dual culture assay against *F. oxysporum*, *F. solani* and *M. phaseolina).***

**Supplementary Table S1. displays the geographical locations and details of the collected samples.**

| **S. No.** | **Plant Name** | **Location** | **GPS Coordinates** | **Sampling Season/Month** | **Environmental Temperature** | **Sample Type** | **No. of Samples Collected** | **No. of Bacterial Isolates** | **Isolate Codes** |
| --- | --- | --- | --- | --- | --- | --- | --- | --- | --- |
| 1 | *Myrica esculenta* | R.I. colony, Panthaghati, Shimla District, Himachal Pradesh | 31.08°N, 77.16° E | February 2025 | 10 ± 2°C | Rhizospheric soil 1 | 1 | 3 | MC1, MC2, MC3 |
|  |  |  | 31.09°N, 77.18° E |  |  | Rhizospheric soil 2 | 1 | 2 | MC4, MC5 |
|  |  |  | 31.10°N, 77.19° E |  |  | Rhizospheric soil 3 | 1 | 4 | MC6, MC7, MC8, MC9 |
| 2 | *Carissa carandas* | Dhundan village, Solan District, Himachal Pradesh | 31.14°N, 76.90° E | February 2025 | 17°C ± 2°C | Rhizospheric soil | 1 | 6 | SR1, SR2, SR3, SR4, SR5, SR6 |
|  |  |  |  |  |  | Leaves | 1 | 2 | K1I, K1II |
|  |  |  |  |  |  | Stem | 1 | 2 | K1III, K1IV |
| 3 | *Pyrus pashia* | Piplughat, Solan District, Himachal Pradesh | 31.02° N, 77.02° E | February 2025 | 19°C± 2°C | Rhizospheric soil | 1 | 4 | SR7, SR8, SR9, SR10 |
|  |  |  |  |  |  | Leaves | 1 | 2 | K2I, K2II |
|  |  |  |  |  |  | Stem | 1 | 2 | K2III, K2IV |

**Supplementary data 1. 16S rDNA consensus sequence**The consensus nucleotide sequence obtained from isolate MC3ʼs 16S rRNA gene is presented below in FASTA format:

*>Lelliottia aquatilis* strain MC3 16S ribosomal RNA gene, partial sequence.

GCGCTGGCGGCAGGCATAACACACGCAAGTCGAGCGGTAGCACAGAGAGCTTGCTTTCGGGTGACGAGCGGCGGACGGGTGAGTAATGTCTGGGAAACTGCCTGATGGAGGGGGATAACTACTGGAAACGGTAGCTAATACCGCATAACGTCGCAAGACCAAAGAGGGGGACCTTCGGGCCTCTTGCCATCACATGTGCCCAGATGGGATTAGCTAGTAGGTGGGGTAATGGCTCACCTAGGCGACAATCCCTAGCTGGGCTGAGAGGATGACCACCCACACTGGAACTGAGACACGGTCCACACTCCTACAGGAGGCAGCAGTGGGGAATATTGCACAATGGGCGCAAGCCTGATGCACCCATGCCGCGTGTATGAAAAAAGCCTTCGGGTTGTAAAGTACTTTCACCGAGGAGGAAAGCATTGTGGTTAATAACCCCAGTGATTGACGTTACTCGCACAAAAAACACCGGCTAACTCCCTGCCACCACCCGCGGTAATACAGAGGGTGCAAGCGTTAATCGCAATTACTGGGCGTAAAGCGCACGCACGCGGTCTGTCAAGTCGGATGTGAAATCCCCGGGCTCAACCTGGGAACTGCATTCGAAACTGGCAGGCTAGAGTCTTGTAGAGGGGGGTAGAATTCCAGGTGTAGCGGTGAAATGCGTAGAGATCTGGAGGAATACCGGTGGCGAAAGCGGCCCCCTGGACAAAGACTGACGCTCAGGTGCGAAAGCGTGGGGAGCAAACAGGATTAGATACCCTGGTAGTCCACGCCGTAAACGATGTCGACTTGGAGGTTGTTCCCTTGAGGAGTGGCTTCCGGAGCTAACACGTTAAATCGACCGCCTGGGGAGTACAGCCGCAAAGTTAAAACTCAAATGAATTGACGGGGGCCCGCACAAGCGGTGGAGCATGTGGTTTAATTCGATGCAACGCGAAGAACCTTACCTACTCTTGACATCCACGGAATTTAGCAGAGATGCTTTAGTGCCTTCGGGAACCGTGAGACAGGTGCTGCATGGCTGTCGTCAGCTCGTGTTGTGAAATGTTGGGTTAAGTCCCGCAACGAGCGCAACCCTTATCCTTTGTTGCCAGCGGTTCGGCCGGGAACTCAAAGGAGACTGCCAGTGATAAACTGGAGGAAGGTGGGGATGACGTCAAGTCATCATGGCCCTTACGAGTAGGGCTACACACGTGCTACAATGGCGTATACAAAGAGAAGCGACCTCGCGAGAGCAAGCGGACCTCATAAAGTACGTCGTAGTCCGGATTGGAGTCTGCAACTCGACTCCATGAAGTCGGAATCGCTAGTAATCGTAGATCAGAATGCTACGGTGAATACGTTCCCGGGCCTTGTACACACCGCCCGTCACACCATGGGAGTGGGTTGCAAAAGAAGTAGGTAGCTTAACCTTCGGGAGGGCGCTTACCACTTTGTGATTCATGACTG

**Supplementary data 2. Representative images of the dual culture assay for biocontrol activity by potential bacterial isolates.**

**SR2	SR3	SR7	Control (*F. oxysporum*)**

**K1III	K1IV	K2III	Control (*F. oxysporum*)**

**MC3	MC6	MC4	Control (*F. oxysporum*)**

**SR2	SR4	SR7	Control (*F. solani*)**

**MC3	MC6	MC4	Control (*F. solani*)**

**K1III	K1IV	K2III	Control (*F. solani*)**

**SR2	SR3	SR7	Control (*M. phaseolina*)**

**MC3	MC6	MC4	Control (*M. phaseolina*)**

**K1III	K1IV	K2III	Control (*M. phaseolina*)**
